# Supplementary material for: Age-related constraints on the spatial geometry of the brain
Source: Nat Commun. 2025 Sep 29;16:8613. doi: 10.1038/s41467-025-63628-3 (PMC12480501; doi:10.1038/s41467-025-63628-3)
Supplement: Supplementary file 1 — Supplementary Information [file 41467_2025_63628_MOESM1_ESM.pdf]

SUPPLEMENTARY TABLES/ FIGURES

Supplementary Table 1. Breakdown of age groups for contrasts.

Group	OASIS			Cam-CAN		
	Age Range	Mean Age	N	Age Range	Mean Age	N
1	42.7-59.9	54.3	249	30.0-37.0	34.0	80
2	60.0-65.8	63.2	258	38.0-44.0	41.1	65
3	65.9-68.7	67.4	244	45.0-51.0	47.9	74
4	68.8-71.5	70.1	251	52.0-58.0	54.9	71
5	71.6-74.1	72.9	249	59.0-65.0	62.1	68
6	74.2-77.5	75.8	247	66.0-71.0	68.6	67
7	77.6-81.7	79.5	255	72.0-79.0	76.1	82
8	81.8-97.1	85.9	231	80.0-88.0	82.7	57

Eight groups with N numbers of scans were created across each dataset.

Supplementary Table 2. Correlations when comparing results using 20x20 to 15x15 and 20x20 to 30x30.

Variable	Correlation(400,225)	Correlation(400,900)
Continuous Age[1]	0.848	0.975
Age Group[1]	0.786	0.941
Clinical Status[2]	0.662	0.949
Episodic Memory[3]	0.754	0.899
Executive Function[3]	0.750	0.861
Working Memory[3]	0.695	0.822

Results for correlations computed when examining 15x15 (225), 20x20 (400), and 30x30 (900) data points in whole brain analysis. Lower correlation values in the second column suggest that 400 data points includes more detailed information about the brain than 225 data points. However, high correlation values in the third column signal suggest that increasing to 900 data points includes about the same amount of information examined when using 400 points.

Supplementary Table 3. Demographics of the Cam-CAN replication sample.

Demographic Variable	Cam-CAN, N = 564
Age (years, *M* ± *SD*)	54.01 ± 18.43; Range: 30-88
Sex (female: n, %)	325 (50.5%)
Education (n, %)	
College or university degree or higher	334 (59.2%)
A levels/ AS levels or equivalent	337 (59.8%)
O levels/ GCSEs or equivalent	418 (74.1%)
CSEs or equivalent	61 (10.8%)
NVQ or HND or HNC or equivalent	80 (14.2%)
Other professional qualifications (e.g.; nursing, teaching)	221 (39.2%)
Race/Ethnicity (n, %)	
White	543 (96.3%)
Mixed	6 (1.1%)
Asian or Asian British	8 (1.4%)
Black or Black British	2 (0.0%)
Chinese	1 (0.0%)
Other ethnic group	3 (0.1%)
Fluid Intelligence (raw score, *M* ± *SD*)	31.98 ± 6.71

Supplementary Table 4. Statistics for replication of key results in the Cam-CAN dataset.

Contrast	Sum of Squares	Mean Square	Num DF	Den DF	F	p
**Global Distances**						
Continuous Age[1]	27390.08	338.15	81	174580.10	3.04	1.47e-18
Age Group[1]	73619.13	129.84	567	173985.73	1.17	3.38e-03
Fluid Intelligence[2]	15476.20	191.06	81	170656.80	1.71	7.27e-05
**Regional Homologues**						
Continuous Age[1]	6480.78	158.07	41	22773.27	24.22	3.00e-177
Age Group[1]	8509.05	29.65	287	22524.91	4.56	5.48e-125
Fluid Intelligence[2]	3604.50	87.91	41	22248.67	13.20	2.43e-87

All models predicted to global distance along inferior to superior and anterior to posterior gradients, controlling for sex, estimated total intracranial volume, and scan quality. Statistics reflect the three-way interaction between the variable of interest, inferior-superior, and anterior-posterior gradients (Mixed effect regression). [1]Models additionally controlled for clinical status. [2]Model additionally controlled for age. [3]Model additionally controlled for age and clinical status.

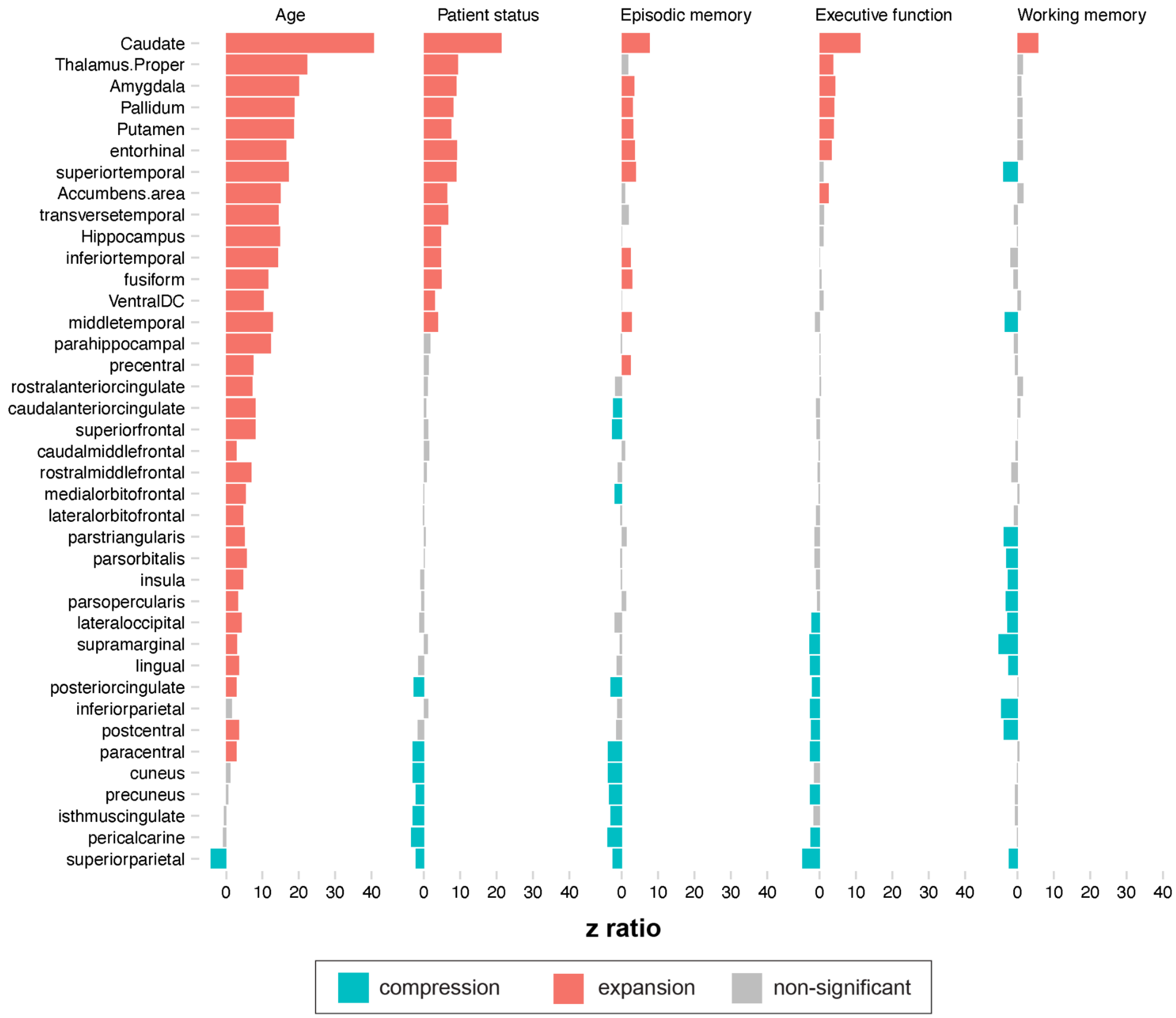

Supplementary Figure 1. Effect sizes of regional homologues results. Effect sizes (z ratio) for significant expansion (red) or compression (blue) of each regional homologue are shown for continuous age, patient status (Clinical Dementia Rating >0), episodic memory, executive function, and working memory. Non-significant effects (after multiple comparison correction) are shown in gray.

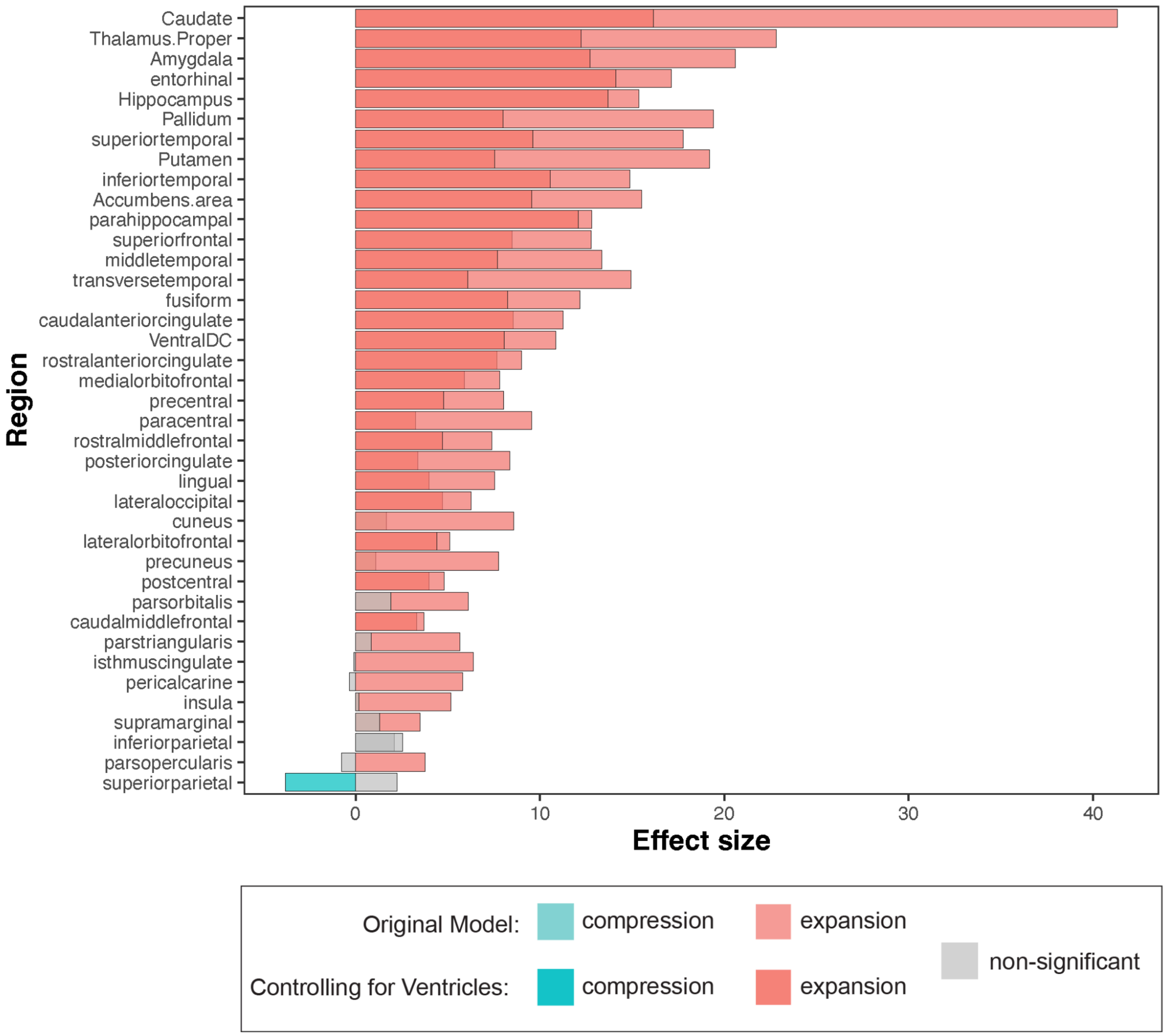

Supplementary Figure 2. Effect sizes of age-related regional homologues results comparing initial models with models additionally controlling for ventricular volume. Lighter shades indicate effect sizes for the original models, and darker shades represent effect sizes after controlling for the effect of ventricular volume in a region-wise manner. Gray indicates a non-significant effect of either expansion or compression.

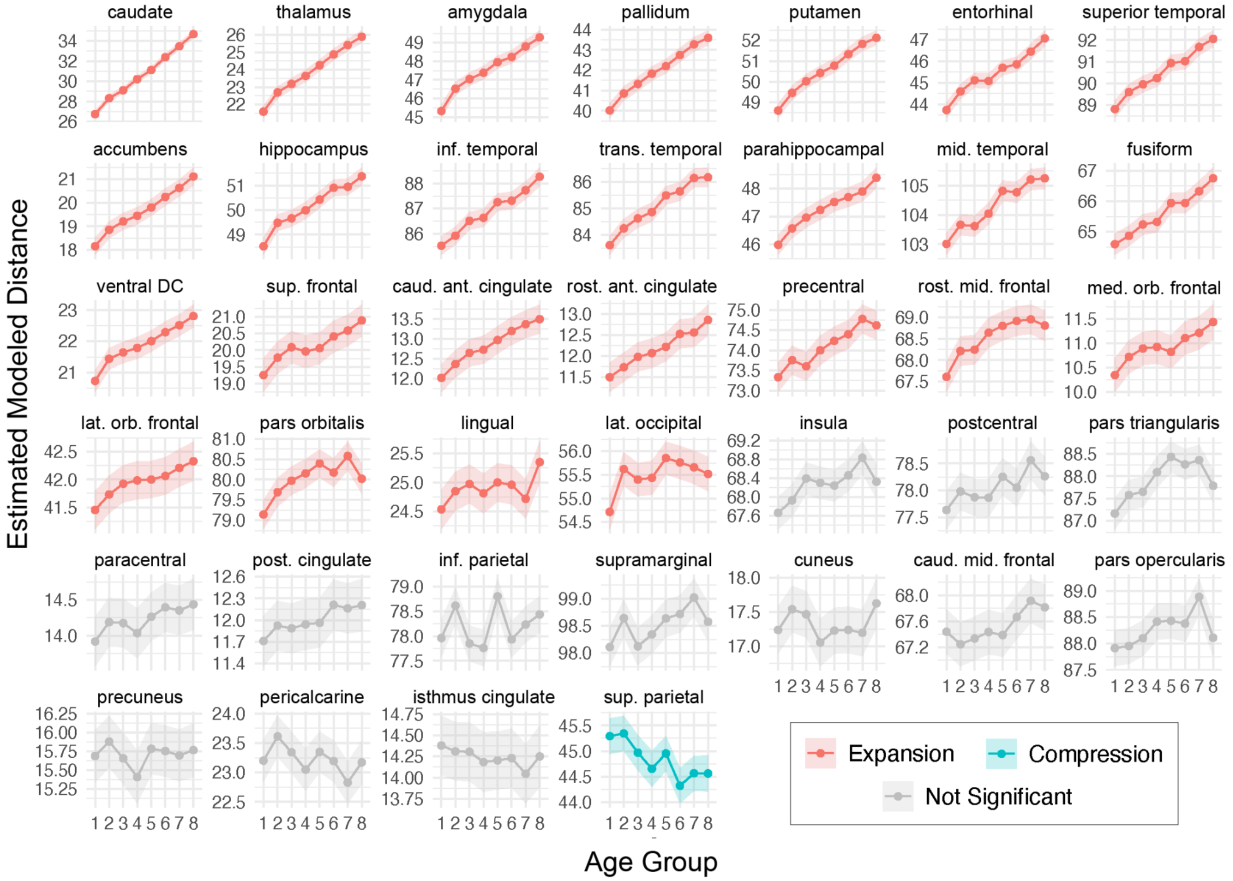

Supplementary Figure 3. Effects of increasing age group on distance between regional homologues. Increasing age group bins (1-8) are shown on the x-axis, while the distance between each regional homologue are shown on the y-axis, allowing for visualization of cross-sectional spatial distance changes. Significant expansion is shown in red, compression in blue, and non-significant (after multiple comparison correction) is shown in gray.Shaded ribbon represents 95% CI.

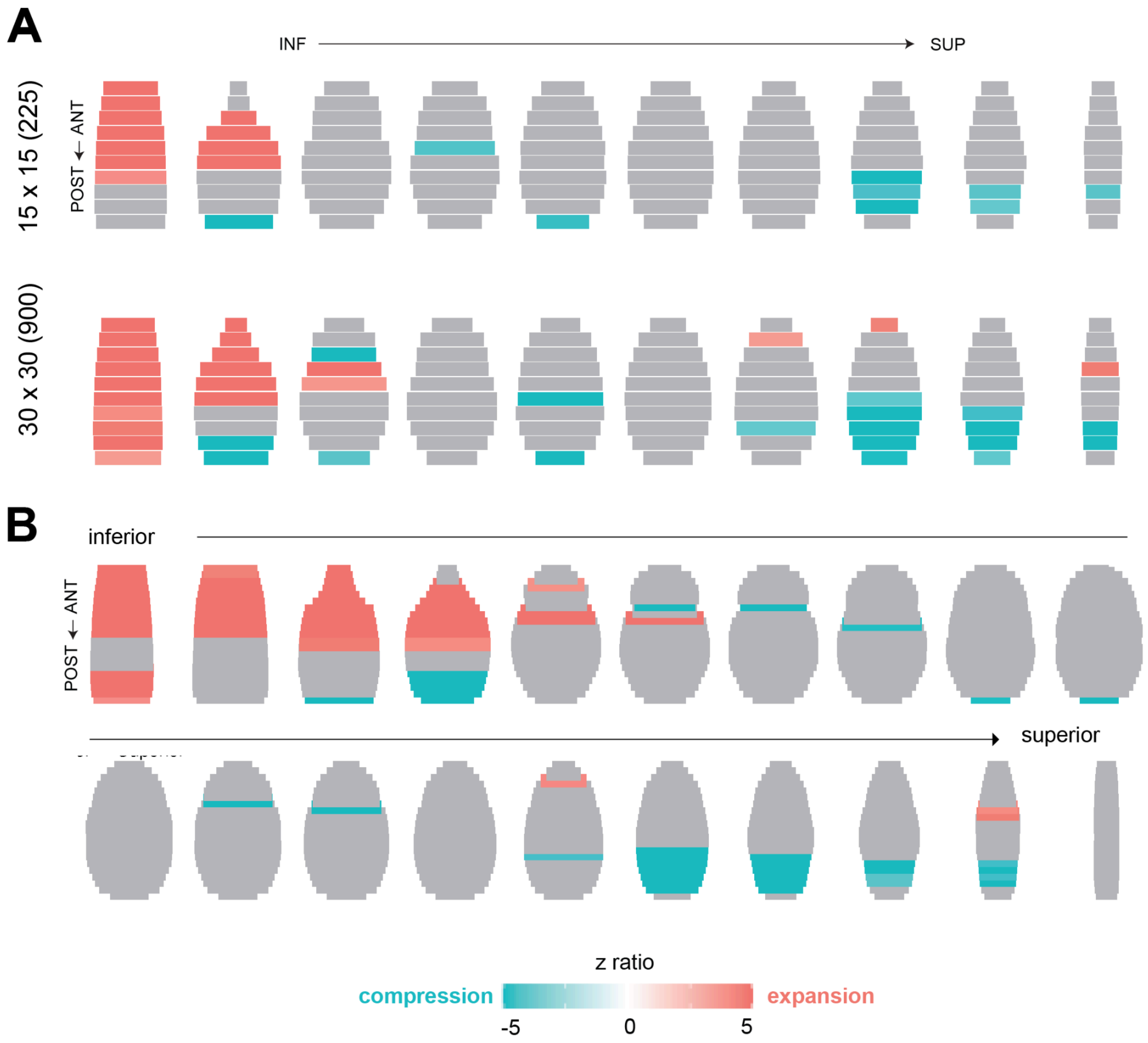

Supplementary Figure 4. Sensitivity analyses varying methodological parameters for the global distance analyses. (A) Patterns of global expansion and compression were similar to initial results (20 x 20 locations, 400 points) when varying the number of points placed along the outer edge of the brain to a smaller (15 x 15 locations, 225 points) or greater (30 x 30 locations, 900 points) number. **(B)** With no downsampling (reducing 20 points to 10 points to facilitate analytic computations) applied in the statistical models, we observed convergent results with our original analyses.

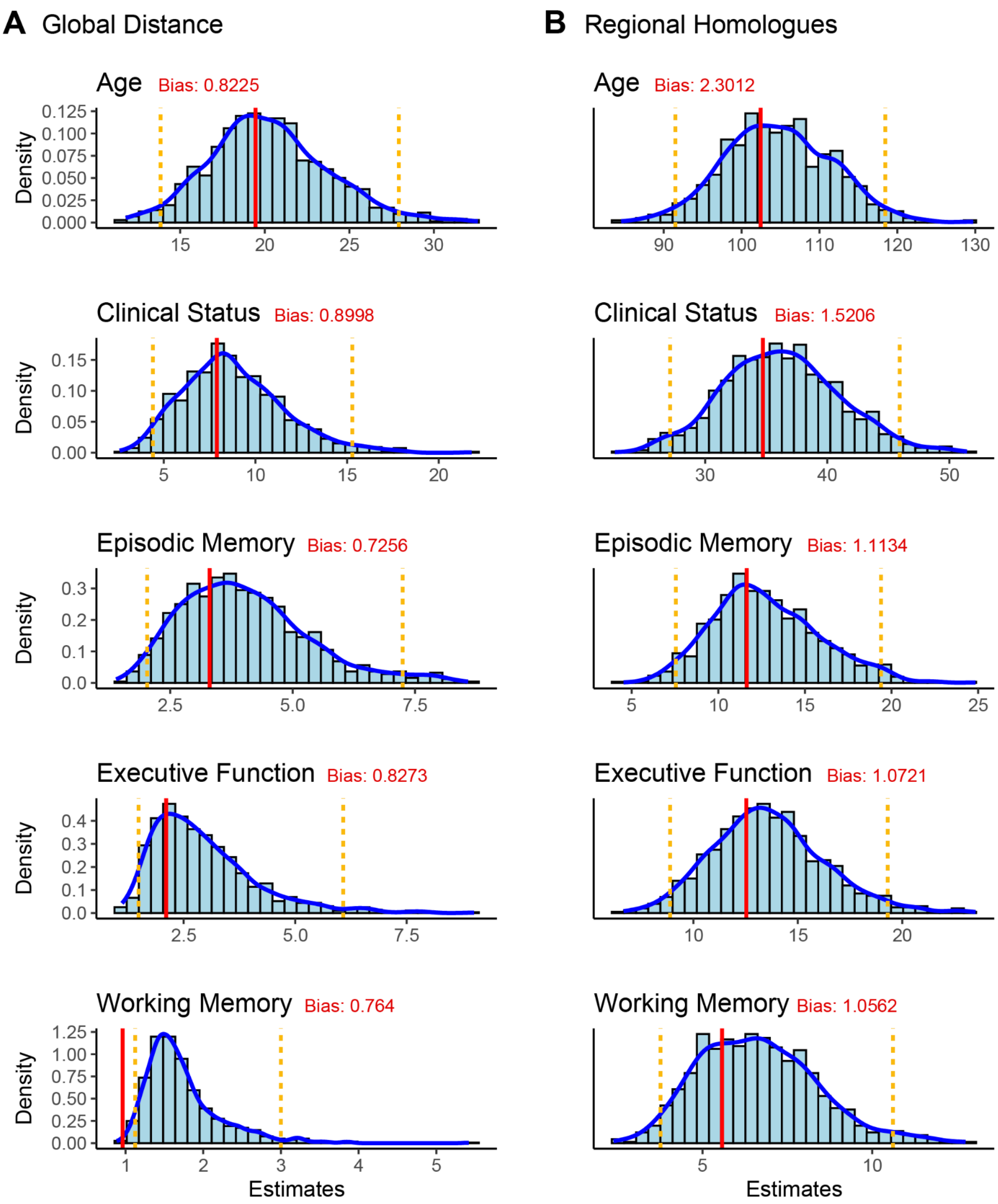

Supplementary Figure 5. Sensitivity analyses demonstrating internal validation of major results. To test internal validity of our analyses, we performed bootstrap analysis with replacement for all primary analyses within **(A)** global distance and **(B)** regional homologues (age, clinical status, and cognitive domains). Histograms representing bootstrapped results demonstrated no significant bias in overestimation of the observed results, as the observed effect size (red solid line) fell within the 95% confidence interval (orange dotted line).

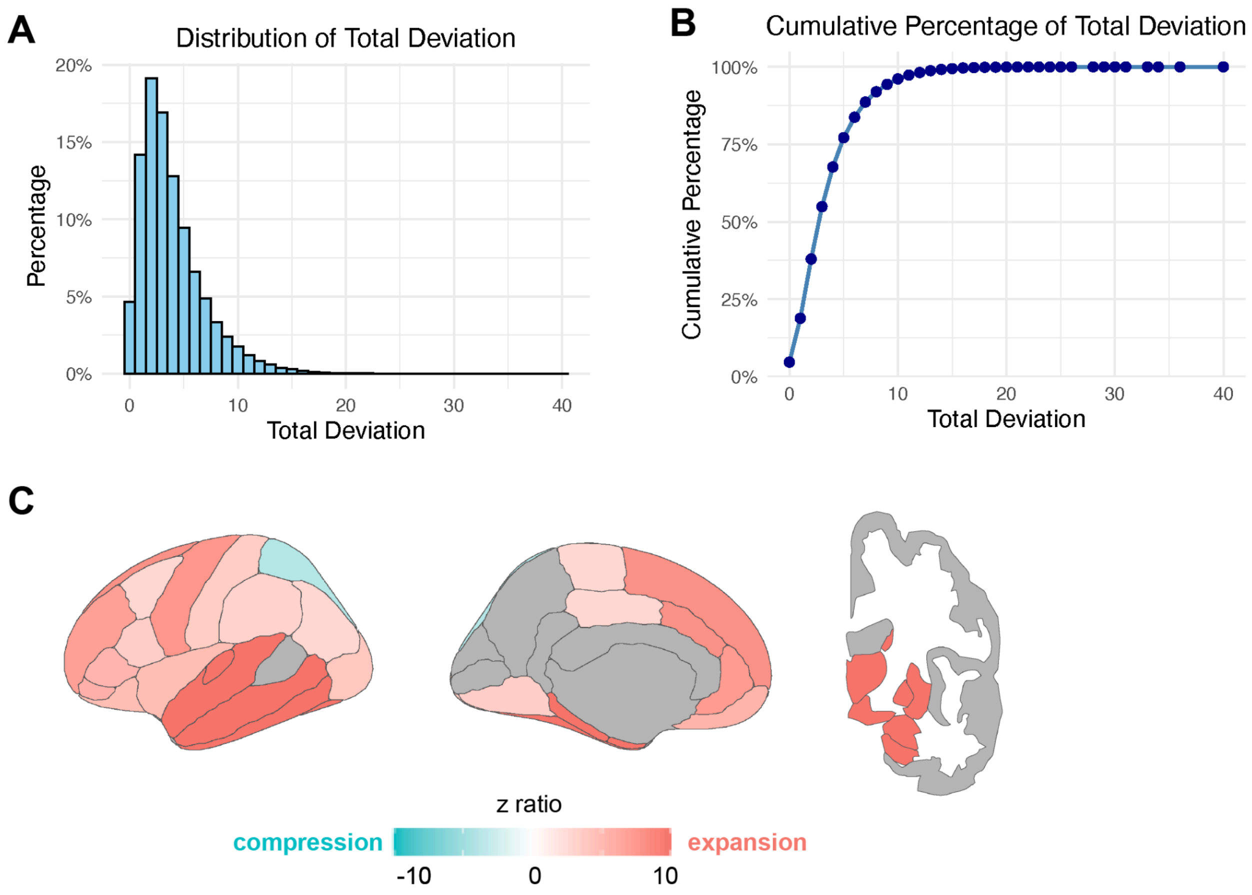

Supplementary Figure 6. Evaluation of deviation of straight line between homologous regions on results. A, Histogram demonstrating the overall total deviation from a straight line was relatively minor (i.e., majority of data had deviation < 4). **B**, The distribution of the deviation, shown as cumulative percentage, demonstrated few outliers. **C**, Visualization of continuous age effects when restricting analyses to homologue pairs with deviation scores of <10.

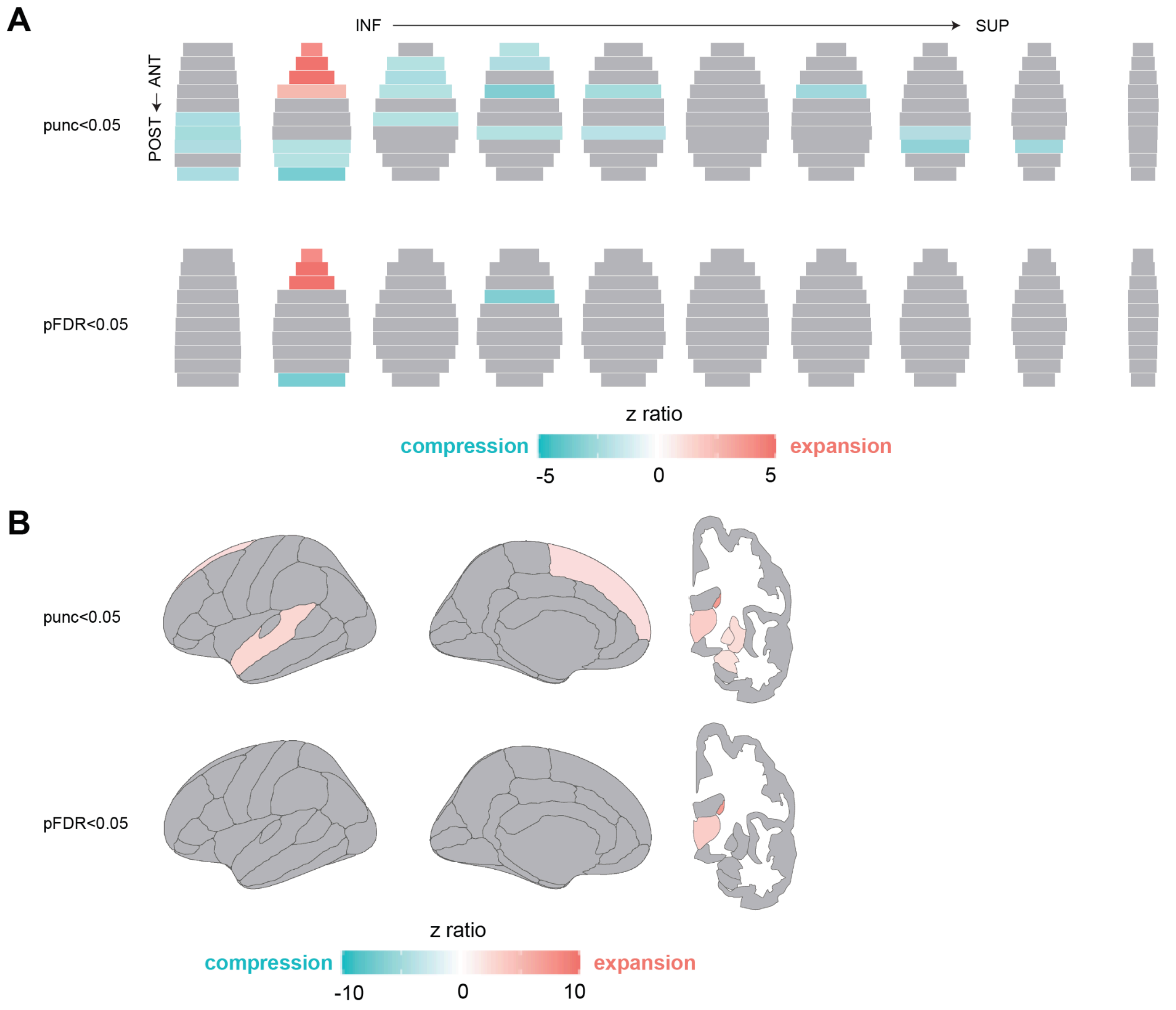

Supplementary Figure 7. Sensitivity analyses demonstrating exploratory longitudinal within-subject change in brain geometry. Within OASIS3, we performed post-hoc construction of a longitudinal dataset by maximizing the number of years between baseline and follow-up scans yielded 499 individuals whose mean age was 68.5 years old (SD = 9.6 years) at baseline and 73.5 years old (SD = 8.5 years) at follow-up. Models of longitudinal change in **(A)** global distance and **(B)** regional homologue expansion and compression were in line with cross-sectional results. Results are presented both corrected (pFDR<0.05) and uncorrected (punc<0.05) for multiple comparisons to better represent the overall pattern of expansion and compression to compare with the cross-sectional results.

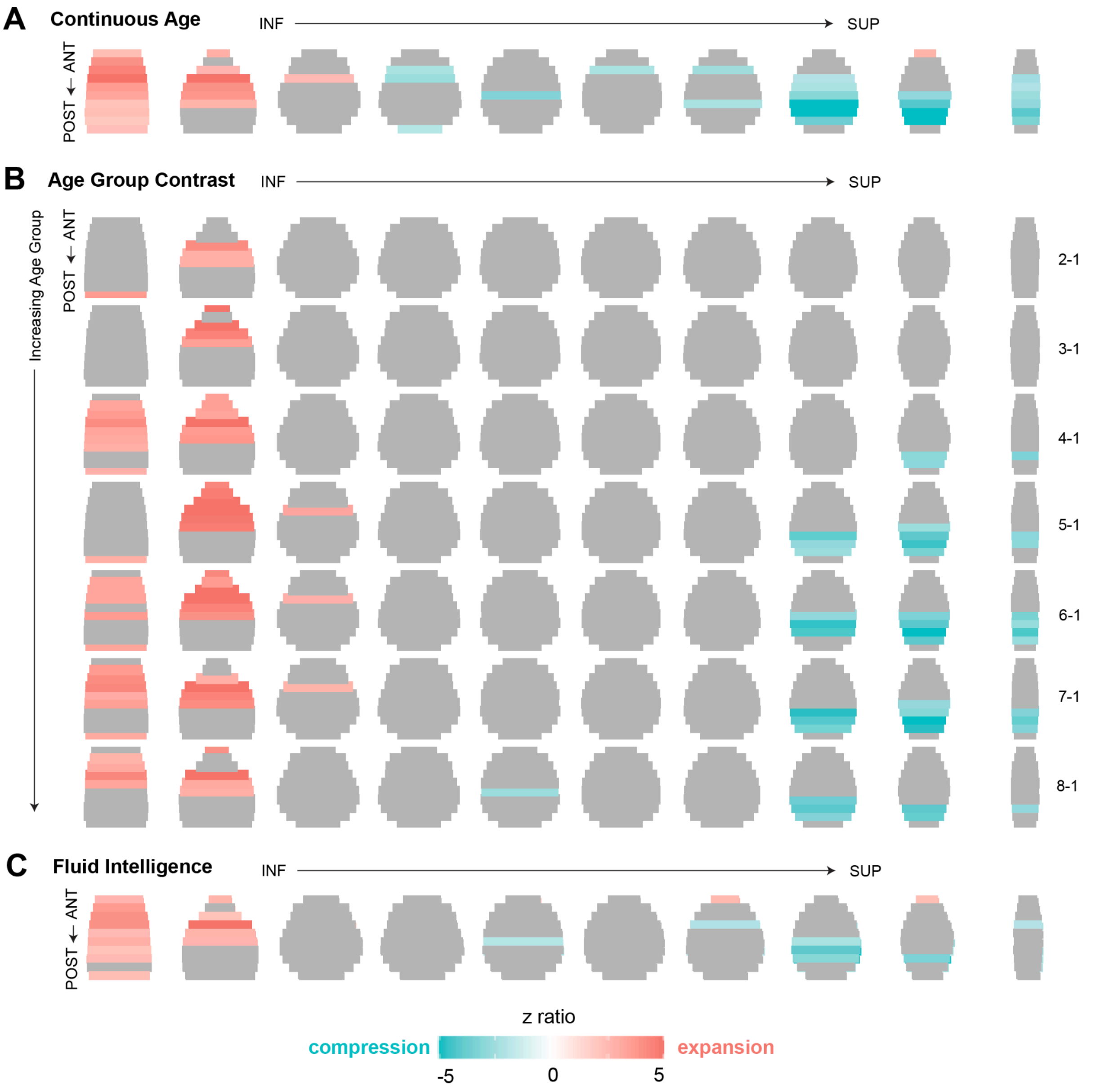

Supplementary Figure 8. Replication of age- and cognition- related whole brain distance results in the Cam-CAN dataset. Within Cam-CAN, increasing age **(A)** and increasing age group contrasts **(B)** was associated with inferior-anterior expansion and superior-posterior compression, similar to those observed within the primary OASIS dataset. **(C)** Fluid intelligence, a general measure of cognition, showed expansion and compression patterns similar to that of aging, even when age was controlled for in the model.

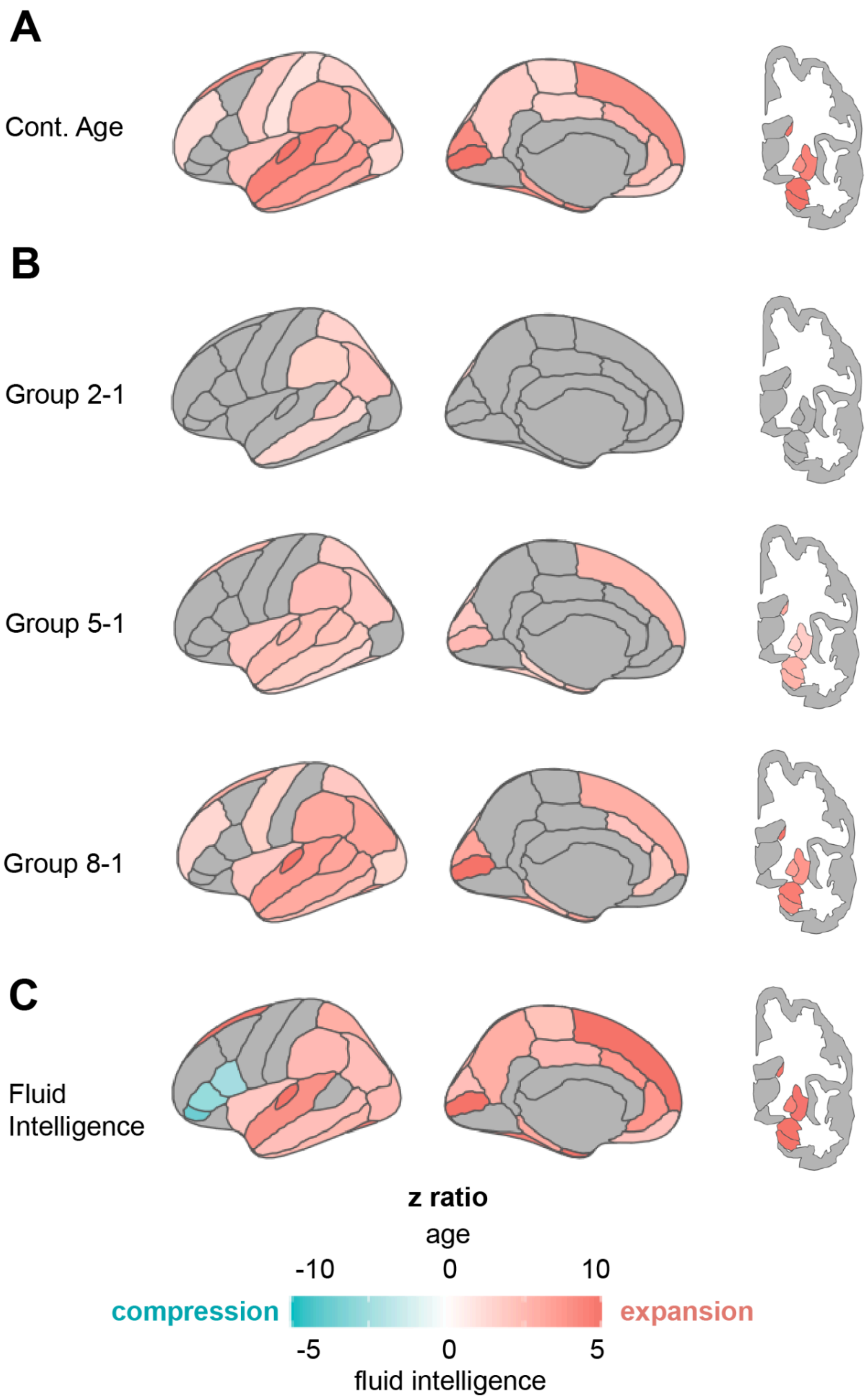

Supplementary Figure 9. Replication of age- and cognitive-related changes in distances between regional homologues in the Cam-CAN dataset. Effects of increasing continuous age **(A)** and increasing age group comparisons **(B)** predominantly showed expansion effects, consistent with results from the OASIS dataset. **(C)** Fluid intelligence was associated with expansion throughout most of the brain, and compression in lateral frontal regions, when controlling for age.
