# Supplementary material for: Age-related constraints on the spatial geometry of the brain
Source: Nat Commun. 2025 Sep 29;16:8613. doi: 10.1038/s41467-025-63628-3 (PMC12480501; doi:10.1038/s41467-025-63628-3)
Supplement: Supplementary file 2 — Description of Additional Supplementary Files [file 41467_2025_63628_MOESM2_ESM.pdf]

Description of Additional Supplementary Files:

Supplementary Movie 1: 3D representation of the continuous age results

Supplementary Movie 2: 3D representation of the age group contrast
