# Supplementary material for: Age-related constraints on the spatial geometry of the brain
Source: Nat Commun. 2025 Sep 29;16:8613. doi: 10.1038/s41467-025-63628-3 (PMC12480501; doi:10.1038/s41467-025-63628-3)
Supplement: Supplementary file 5 — Reporting Summary [file 41467_2025_63628_MOESM5_ESM.pdf]

nature portfolio

Corresponding author(s): Niels Janssen

Last updated by author(s): Jun 22, 2025

Reporting Summary

Nature Portfolio wishes to improve the reproducibility of the work that we publish. This form provides structure for consistency and transparency in reporting. For further information on Nature Portfolio policies, see our Editorial Policies and the Editorial Policy Checklist.

Statistics

For all statistical analyses, confirm that the following items are present in the figure legend, table legend, main text, or Methods section.

n/a	Confirmed	
☐	☒	The exact sample size (n) for each experimental group/condition, given as a discrete number and unit of measurement
☐	☒	A statement on whether measurements were taken from distinct samples or whether the same sample was measured repeatedly
☐	☒	The statistical test(s) used AND whether they are one- or two-sided<br>*Only common tests should be described solely by name; describe more complex techniques in the Methods section.*
☐	☒	A description of all covariates tested
☐	☒	A description of any assumptions or corrections, such as tests of normality and adjustment for multiple comparisons
☐	☒	A full description of the statistical parameters including central tendency (e.g. means) or other basic estimates (e.g. regression coefficient) AND variation (e.g. standard deviation) or associated estimates of uncertainty (e.g. confidence intervals)
☐	☒	For null hypothesis testing, the test statistic (e.g. F, t, r) with confidence intervals, effect sizes, degrees of freedom and P value noted<br>*Give P values as exact values whenever suitable.*
☒	☐	For Bayesian analysis, information on the choice of priors and Markov chain Monte Carlo settings
☐	☒	For hierarchical and complex designs, identification of the appropriate level for tests and full reporting of outcomes
☐	☒	Estimates of effect sizes (e.g. Cohen's d, Pearson's r), indicating how they were calculated

Our web collection on statistics for biologists contains articles on many of the points above.

Software and code

Policy information about availability of computer code

Data collection	no software was used
Data analysis	Bash, FSL , Python, R. All code is available on Code Ocean.

For manuscripts utilizing custom algorithms or software that are central to the research but not yet described in published literature, software must be made available to editors and reviewers. We strongly encourage code deposition in a community repository (e.g. GitHub). See the Nature Portfolio guidelines for submitting code & software for further information.

Data

Policy information about availability of data

All manuscripts must include a data availability statement. This statement should provide the following information, where applicable:

- Accession codes, unique identifiers, or web links for publicly available datasets
- A description of any restrictions on data availability
- For clinical datasets or third party data, please ensure that the statement adheres to our policy

The data that support the findings of this study are available from OASIS and CAM-CAN upon registration and compliance with their data usage agreements.

Research involving human participants, their data, or biological material

Policy information about studies with human participants or human data. See also policy information about sex, gender (identity/presentation), and sexual orientation and race, ethnicity and racism.

Reporting on sex and gender	Sex (male, female) was included as a covariate in all statistical models. We report the main effect of sex on our effects of interest for transparent statistical reporting.
Reporting on race, ethnicity, or other socially relevant groupings	The open access datasets used here were predominantly non-Hispanic white with little diversity, which is a limitation of the current study. Due to the breakdown of race and ethnicity, we were not statistically powered to conduct sub analyses to appropriately test how these factors may have influenced our results. Future work in more diverse samples should answer this important question.
Population characteristics	The sample ranged in age from about 30-90 years across both datasets. Within the OASIS dataset, some participants were considered "cognitively impaired" based upon a Clinical Dementia Rating score of >0. We did not have genotype available, and no participants were in treatment groups.
Recruitment	Recruitment of study participants were performed by the OASIS and CAMCAN study teams - more information is available in the cited parent studies for each open access dataset.
Ethics oversight	The current study used open access data from OASIS and CAMCAN - ethics oversight was performed by each parent study and described in more detail in the methods section and each respective publication

Note that full information on the approval of the study protocol must also be provided in the manuscript.

Field-specific reporting

Please select the one below that is the best fit for your research. If you are not sure, read the appropriate sections before making your selection.

☒ Life sciences ☐ Behavioural & social sciences ☐ Ecological, evolutionary & environmental sciences

For a reference copy of the document with all sections, see nature.com/documents/nr-reporting-summary-flat.pdf

Life sciences study design

All studies must disclose on these points even when the disclosure is negative.

Sample size	No sample size calculation was performed. We selected the largest publicly available dataset (OASIS3) that contained the variables required for our study.
Data exclusions	Data were excluded when relevant study variables were unavailable and when data did not pass quality controls (scan quality, region-wise dependent measure outside interquartile range).
Replication	Reproducibility of the results obtained from the OASIS3 dataset were ascertained through 1) a within sample longitudinal replication, 2) a within-sample bootstrap validation, 3) a replication to a new sample (Cam-Can).
Randomization	Allocation was not random. Regression analyses included co-variates for gender, age, clinical status, scan quality, total intracranial volume, regional atrophy, and ventricle size when required.
Blinding	Blinding was not relevant to our study since we worked with existing datasets and were not directly involved in data acquisition.

Reporting for specific materials, systems and methods

We require information from authors about some types of materials, experimental systems and methods used in many studies. Here, indicate whether each material, system or method listed is relevant to your study. If you are not sure if a list item applies to your research, read the appropriate section before selecting a response.

Materials & experimental systems

n/a	Involved in the study
☒	☐ Antibodies
☒	☐ Eukaryotic cell lines
☒	☐ Palaeontology and archaeology
☒	☐ Animals and other organisms
☒	☐ Clinical data
☒	☐ Dual use research of concern
☒	☐ Plants

Methods

n/a	Involved in the study
☒	☐ ChIP-seq
☒	☐ Flow cytometry
☐	☒ MRI-based neuroimaging

Plants

Seed stocks

Report on the source of all seed stocks or other plant material used. If applicable, state the seed stock centre and catalogue number. If plant specimens were collected from the field, describe the collection location, date and sampling procedures.

Novel plant genotypes

Describe the methods by which all novel plant genotypes were produced. This includes those generated by transgenic approaches, gene editing, chemical/radiation-based mutagenesis and hybridization. For transgenic lines, describe the transformation method, the number of independent lines analyzed and the generation upon which experiments were performed. For gene-edited lines, describe the editor used, the endogenous sequence targeted for editing, the targeting guide RNA sequence (if applicable) and how the editor was applied.

Authentication

Describe any authentication procedures for each seed stock used or novel genotype generated. Describe any experiments used to assess the effect of a mutation and, where applicable, how potential secondary effects (e.g. second site T-DNA insertions, mosiacism, off-target gene editing) were examined.

Magnetic resonance imaging

Experimental design

Design type

Indicate task or resting state; event-related or block design.

Design specifications

Specify the number of blocks, trials or experimental units per session and/or subject, and specify the length of each trial or block (if trials are blocked) and interval between trials.

Behavioral performance measures

State number and/or type of variables recorded (e.g. correct button press, response time) and what statistics were used to establish that the subjects were performing the task as expected (e.g. mean, range, and/or standard deviation across subjects).

Acquisition

Imaging type(s): structural

Field strength: 3T

Sequence & imaging parameters: The T1w images were acquired using a 3DMPRAGE protocol TI/TR/TE: 1000/2400/3.08 ms, flip angle = 8º, resulting in 1 mm isotropic voxels. In addition, T2w images were acquired using a 3D SPC protocol: TR/TE: 3200/0.455 ms, flip angle = 120º, resulting in 1 mm isotropic voxels.

Area of acquisition: whole brain

Diffusion MRI ☐ Used ☒ Not used

Preprocessing

Preprocessing software: Human Connectome Project minimal preprocessing pipeline v4.1.3

Normalization: Data were not normalized. Normalization would preclude finding an association between brain shape and aging.

Normalization template: Data were not normalized.

Noise and artifact removal: 1. averaging repeated T1w and T2w acquisitions if such exist; 2. Perform alignment to ACPC axes in native structural space and undistort images with GRE fieldmaps; 3. Perform an initial robust brain extraction using FNIRT; 4. Align the T1w and T2w images; 5. Perform bias field correction.

Volume censoring

Define your software and/or method and criteria for volume censoring, and state the extent of such censoring.

Statistical modeling & inference

Model type and settings	All statistical modeling was performed in R (v4.1.2) using the package lme4 (v1.1-31) for mixed effect regression modeling. We converted the output of the extensive regression tables into more comprehensive ANOVA tables with p-values using the Saitherwait approximation using the lmerTest package (v3.1-3). Whenever relevant interactions were significant ($p < 0.05$) post-hoc tests with bonferroni corrections for multiple comparisons were performed using the emmeans package (v1.8.4-1).
Effect(s) tested	We tested for the effect of age (continuous, or by age-group), clinical status (patient vs control), episodic memory (continuous or by group), working memory (continuous or by group), and executive function (continuous or by group).

Specify type of analysis: ☒ Whole brain ☐ ROI-based ☐ Both

Statistic type for inference<br>(See Eklund et al. 2016)	*Specify voxel-wise or cluster-wise and report all relevant parameters for cluster-wise methods.*
Correction	All post-hoc tests were bonferroni corrected, except the longitudinal analyses which were FDR corrected.

Models & analysis

n/a	Involved in the study
☒	☐ Functional and/or effective connectivity
☒	☐ Graph analysis
☒	☐ Multivariate modeling or predictive analysis
